# Effect of substrate on the proliferation of *Myxobolus cerebralis* in the mitochondrial lineages of the *Tubifex tubifex* host

**DOI:** 10.1007/s00436-022-07587-4

**Published:** 2022-07-27

**Authors:** Dolores V. Baxa, R. Barry Nehring

**Affiliations:** 1 Department of Medicine and Epidemiology, School of Veterinary Medicine, University of California, Davis, CA 95616 USA; 2 Department of Anatomy, Physiology, and Cell Biology, School of Veterinary Medicine, University of California, Davis, CA 95616 USA; 3 Colorado Division of Parks and Wildlife, 2300 South Townsend Avenue, Montrose, CO 81401 USA

**Keywords:** Whirling disease, *Tubifex tubifex*, Triactinomyxon, Substrate

## Abstract

The study goal was to examine the effects of sand and mud on the propagation of *Myxobolus cerebralis*, the whirling disease agent, in four mitochondrial 16S ribosomal DNA lineages (I, III, V, VI) of its oligochaete host, *Tubifex tubifex (Tt)*. In all the lineage groups held continuously in either substrate (non-shifted) or transferred from sand to mud (shifted), substrate influenced parasite proliferation only in lineage III. Sporogenesis and release of triactinomyxon spores (TAMs) were more prevalent in lineage III *Tt* in mud compared to sand. Low-infection prevalence and lack of parasite development in lineage I is associated with the greater number of resistant worms and were not affected by substrate type. Substrate did not impact *Tt* from lineages V and VI that failed to develop any parasite stages in either substrate even after shifting from sand to mud. The relationship between the microbial community in the substrate and parasite proliferation in lineage III was described but not analyzed due to small sample size. Substrate-associated bacteria were hypothesized as essential dietary source for the oligochaete host feeding selectively on fine (mud)-microflora. Progeny was produced by all lineage groups shifted to mud with disparate survival profiles in lineage V and VI and high mortalities in lineage III. Our study demonstrates that substrate type can alter parasite proliferation in lineage III. Conversely, parasite development and infectivity were not altered in lineage V and VI that are refractory to the parasite nor among the more resistant phenotypes (I), regardless of substrate type.

**Supplementary Information:**

The online version contains supplementary material available at 10.1007/s00436-022-07587-4.

## Introduction

The myxozoan *Myxobolus cerebralis* is the causative agent of whirling disease (WD) in salmonid fish (Hofer 1903) involving two obligatory hosts, a susceptible salmonid and the aquatic tubificid oligochaete (hereafter referred to as oligochaete) *Tubifex tubifex* (*Tt*) (Markiw and Wolf 1983; Wolf and Markiw 1984; Wolf et al. 1986). Different stages of the myxozoan develop in both hosts that release morphologically distinct spores (Wolf and Markiw 1984; El-Matbouli et al. 1995; El-Matbouli and Hoffmann 1998; Hedrick and El-Matbouli 2002). Salmonids produce myxospores infectious to the *Tt* host, which in turn produce the actinosporean triactinomyxon spores (TAMs) that infect salmonid fishes (El-Matbouli et al. 1995; Kerans and Zale 2002; Hedrick and El-Matbouli 2002; Gilbert and Granath 2003; Sarker et al. 2015). While several salmonid species are susceptible to *M. cerebralis* (MacConnell and Vincent 2002), *Tt* is the only oligochaete known to host the parasite (Markiw and Wolf 1983; Wolf and Markiw 1984; Wolf et al. 1986; El-Matbouli and Hoffmann 1998; Kerans et al. 2004). Recently, *M. cerebralis* has been detected in non-*Tt* suggesting the potential role of another oligochaete in the life cycle of the parasite (Ksepka et al. 2021).

*Tubifex tubifex* is genetically diverse (Anlauf and Neumann 1997; Sturmbauer et al. 1999) encompassing six mitochondrial ribosomal DNA (mt rDNA) strains that differ in tolerance to environmental variables such as cadmium (Sturmbauer et al. 1999) or vary in susceptibility to experimental infection with *M. cerebralis* (Beauchamp et al. 2001). Susceptible *Tt* release high TAM numbers that vary widely between worm populations (e.g., Steinbach-Elwell et al. 2006; Arsan and Bartholomew 2008; Baxa et al. 2008; Rasmussen et al. 2008, Hallett et al. 2009) while resistant *Tt* do not release TAMs or are refractory to infections following exposure to myxospores (Beauchamp et al. 2005; DuBey et al. 2005; Steinbach-Elwell et al. 2006). Resistant worms are dominant in lineage I (Beauchamp et al. 2006; Arsan et al. 2007a; Nehring et al. 2013, 2014); however, TAM production in susceptible phenotypes can vary across geographic regions (Arsan et al. 2007b; Hallett et al. 2009; Nehring et al. 2014). Lineage III *Tt* has been the most studied, comprising the greatest number of *Tt* susceptible to *M. cerebralis* across North America, although parasite proliferation varies among geographic strains (Stevens et al. 2001; Kerans et al. 2004; Baxa et al. 2008; Rasmussen et al. 2008; Nehring et al. 2013, 2014). Numerous independent studies spanning almost two decades from across western North America have repeatedly demonstrated that *Tt* belonging to lineages V and VI are refractory to *M. cerebralis.* This includes oligochaetes from Alaska (Arsan et al. 2007a), California (Beauchamp et al. 2002; Beauchamp et al. 2006), Colorado (Nehring et al. 2014), New Mexico (DuBey and Caldwell 2004), and Oregon (Hallett et al. 2009). As these studies have shown, the mt 16S rDNA lineages are divergent in susceptibility to infection with *M. cerebralis* and parasite production relative to geographic locality, dose of parasite exposure, genotype composition, and habitat type (Stevens et al. 2001; Beauchamp et al. 2002; Blazer et al. 2003; Dubey and Caldwell 2004; Kerans et al. 2004; Rasmussen et al. 2008; Hallett et al. 2009). Furthermore, differences in parasite proliferation in the *Tt* host have been attributed to contributions of key environmental factors such as water temperature (El-Matbouli et al. 1999c; Kerans and Zale 2002; Blazer et al. 2003; Kerans et al. 2005), confounding environmental features (Kaeser et al. 2006), and complex habitat components (Eby et al. 2015). Altered habitats have also been reported to influence WD propagation (Thompson et al. 2002; Thompson 2011; Granath 2014; Richey et al. 2018).

Since the early 1990s, numerous studies have demonstrated that *M. cerebralis* poses a threat to the persistence of wild trout (*Oncorhynchus* spp.) across western North America (Vincent 1996; Nehring and Walker 1996; Allendorf et al. 2001; Koel et al. 2006; Granath et al. 2007; Murcia et al. 2015; Nehring et al. 2018; James et al. 2021). As shown above, a large body of work has focused on the intricate two-host life cycle of *M. cerebralis* and a multitude of factors contributing to the establishment of the parasite. One critical factor that sustains parasite persistence and the WD cycle is the abundance of susceptible *Tt* in the environment. Here, we investigated how two substrates influence infection and proliferation of *M. cerebralis* in the different lineages of the *Tt* host. Previous studies have implicated sediment type in predisposing the *Tt* host to WD (Arndt et al. 2002; Blazer et al. 2003; Gilbert and Granath 2003; Krueger et al. 2006; Eby et al. 2015). Our earlier work showed that mud facilitated *M. cerebralis* development in pure clonal lines of TAM producing mt 16S rDNA lineage III from non-TAM-producing (resistant) to TAM-producing (susceptible) phenotypes (Baxa et al. 2006, 2008). As the clonal cultures were lost over time, we modified our experimental design to use the mt 16S rDNA lineages of *Tt* (I, III, V, VI) to address a similar fundamental question whether the invertebrate host genetics and/or substrate type alters *M. cerebralis* infectivity. Hence, the hypothesis in this study is that substrate and *Tt* genotype affect the development and production of *M. cerebralis*.

The overarching goal of the present study is to expand our understanding of substrate effect on *M. cerebralis* development and release of TAMs among the mt 16S rDNA lineages (hereafter referred to as lineage) of the *Tt* host deemed more susceptible (lineage III) or more resistant (lineage I) to the parasite. A secondary objective is to determine whether the lack of parasite development in oligochaetes belonging to resistant lineages (V, VI) can be altered by changes to substrate. Moreover, we aimed to determine the potential relationship between the sediment-associated microflora and parasite proliferation in lineage III *Tt* host most susceptible to *M*. *cerebralis* by assessing their gastrointestinal microflora after holding in sand or mud.

## Methods

### Substrate

Mud and sand were obtained from farms adjacent to California Department of Fish and Wildlife, Rancho Cordova, California. Sand particles were collected from Natoma Aquatic Farm while the silt-mud sediment was from Negro Bar Boat Ramp. Both sites are non-enzootic to *M. cerebralis* or WD. Worms were not encountered from these locations at the time of collection or from the sediments brought to the Fish Health wet laboratory at the University of California, Davis. The size of the sand particles ranged from 0.25 to 1.0 mm while the silt-mud sediment ranged from 2.0 to 7.0 μm based on the Wentworth grain size classification (Day 1965). The size of the sediment particles was roughly estimated using fine test sieves (Sigma Aldrich). Particles that were retained in no. 18 sieve (1.0 mm sieve opening) were designated as sand and particles that passed through the sieve as mud. The sediments were placed in separate buckets, washed several times with flow-through well water, and transferred to aerated plastic containers covered with dechlorinated tap water.

### Source and maintenance of *Tubifex tubifex*

Lineage III oligochaetes were collected from Mt. Whitney, State Fish Hatchery, Independence, California. Worms from this site have consistently been typed as lineage III in our laboratory and in other WD studies. Oligochaetes belonging to lineages I, V, and VI were sourced from river drainages in Colorado and maintained as stock cultures by the Colorado Division of Wildlife Aquatic Research staff in Montrose, Colorado. Bulk cultures of the four lineage groups were maintained in 2-L plastic containers with 400 g sterilized sand, 1 L dechlorinated tap water, and held at a 15 °C incubator at the Fish Health wet laboratory at the University of California, Davis. Well water (15 °C) was replaced (50%) in the stock cultures, and oligochaetes were fed once a week with *Spirulina* (Bio-Marine) and Algamac (Bio-Marine) at 8:2 ratio up to 5.5 g/stock culture.

### Assessing mitochondrial lineages of *Tubifex tubifex*

Prior to myxospore exposures, oligochaetes (*N* = 20/lineage group) were randomly collected from the bulk cultures. Total genomic DNA was extracted from individual oligochaetes using the QIAmp tissue kit (Qiagen), initially screened with the *Tt* specific primers (Beauchamp et al. 2001) and verified with the mt 16S rDNA lineage-specific PCR (Beauchamp et al. 2002). Oligochaetes that were negative for these tests were considered non-*Tt*. Although *Tt* has evolved into extended species complex as found in Europe (Marotta et al. 2014), the mt 16S rDNA lineage-specific PCR (Beauchamp et al. 2001) was considered the most appropriate marker for specific identification of *Tt* strains, thus only four *Tt* lineages could be distinguished in the current study using the assay. The proportion of *Tt* lineage type was calculated as the number of oligochaetes positive for the lineage over the number of oligochaetes examined in each group. Lineage typing was also conducted at 80 days post exposure (pe) and at termination of the study at 6 months as described below in the non-shifted and shifted studies.

### Exposure of *Tubifex tubifex* to myxospores

Oligochaetes were randomly collected from the bulk cultures and held in dechlorinated tap water without substrate for 24 h to match hunger levels. The oligochaetes were then counted into plastic containers (8 × 8 cm) containing 128 ml of sand or mud (2 cm) and covered with 320 ml of well water (5 cm depth above the substrate surface). All oligochaete groups were acclimated in the substrate without feeding for another 24 h prior to exposure to *M. cerebralis* myxospores. Myxospores were obtained from the heads of three laboratory infected rainbow trout (Troutlodge) using the plankton centrifuge method (O’Grodnick 1975). Freshly collected spores were enumerated (average of triplicate counts) using a hemocytometer and added to each group at a dose of 1000 myxospores/oligochaete. At the time of exposure, the water level in the containers with the worms was reduced by 75%, spore suspensions were added, and after 4 h, well water was added to the original level. A 31% water change and one feeding with 1.0–1.5 g of *Spirulina* and Algamac (8:2) were conducted each week. Control groups of oligochaetes for each lineage were set up similarly but did not receive spores.

### Non-shifted study

Oligochaetes were continuously held in either sand or mud until termination of the study at 6 months to assess the effect of substrate on *M. cerebralis* development in *Tt* lineages deemed more susceptible (III) or more resistant (I) to the parasite. The experimental design of the non-shifted groups is summarized in Table S1. Four containers of 50 worms/replicate were used for each lineage group. Three replicates were exposed to *M. cerebralis* myxospores as described above with one unexposed control. Water samples were collected from the two exposed replicates of each lineage for estimating TAM release every 7 to10 days beginning at 80 days up to 6 months pe. These two replicates were not sampled; however, at the end of the study at 6 months, survivors in replicate 1 were assessed for individual TAM release. The third exposed replicate was used for assessment of lineage type (Beauchamp et al. 2002) and *M. cerebralis* DNA by PCR (Andree et al. 1998) at 80 days (*N* = 10) and 6 months. All were analyzed for lineage type and parasite DNA by PCR if there were less than 20 oligochaetes in a group at the end of the study. In situ hybridization (ISH) was used to confirm the parasite DNA (Antonio et al. 1998) at 80 days (*N* = 10). Each of the ten oligochaetes were cut in half; the anterior segments were fixed in 10% neutral buffered formalin and sectioned for ISH while the posterior segments were used for genomic DNA extraction for lineage typing. The unexposed control group of each lineage was also sampled (*N* = 10) to test for parasite DNA (PCR) at termination of the study at 6 months.

### Shifted study

Shifted groups were included to determine whether substrate change can alter TAM production particularly for lineages resistant to *M. cerebralis* (I, V, VI). Exposed worms (*N* = 100/lineage) from the different lineages were held in sand for 60 days after which 50 worms were transferred from sand to mud while the 50 worms were retained in sand (Table S1). The shifting process could have stressed the worms in both substrata potentially disrupting parasite development. Release of TAMs was assessed in water samples of groups retained in sand and from groups transferred from sand to mud every 10 days beginning at 80 days up to 6 months. All oligochaetes were analyzed for lineage type and parasite DNA by PCR if there were less than 20 in a group at the end of the study. Individual TAM release was also confirmed in all remaining worms in sand or mud at the end of the study at 6 months.

The non-shifted and shifted groups were maintained in a 12 °C-incubator equipped with a 50-W fluorescent bulb to provide a photoperiod cycle of 14-h light and 10-h dark. After the weekly water sampling (100 ml) for TAM counts, cooled well water (12 °C) was equally replaced into each container. The oligochaetes in each group were fed once a week with 1.0–1.5 g of *Spirulina* and Algamac (8:2).

### Assessment of infection and production of triactinomyxon spores (TAMs)

The prevalence of infections with *M. cerebralis* in *Tt* lineages in sand or mud was assessed by the presence of the parasite DNA in oligochaetes using a single-round PCR assay (Andree et al. 1998) and a nonradioactive in situ hybridization (ISH) method (Antonio et al. 1998, 1999) all previously developed in our laboratory. The single amplification assay for *M. cerebralis* used the primers Tr 5-16 and Tr 3-17 following the method described by Andree et al. (1998). Although both PCR and ISH can specifically detect all stages of *M. cerebralis* development, the ISH method offers anatomic location of infection (Antonio et al. 1998, 1999) hence used to confirm the parasite DNA in oligochaete tissues detected by PCR. Furthermore, early developmental forms of *M. cerebralis* are difficult to detect in low-grade infections in the oligochaete host where traditional spore extraction method cannot identify developmental stages of the parasite (Antonio et al. 1999). We followed the ISH procedure described in Antonio et al. (1998, 1999) using a cocktail of three probes specific to the myxozoan sequences (Tr), Tr 5-16, Tr 3-16, and Tr 3-17 designed to hybridize to homologous sequences of the small subunit ribosomal DNA (rDNA) sequences of *M. cerebralis* (Andree et al. 1998).

The production of TAMs in *Tt* lineage groups following holding in sand or mud (non-shifted) was assessed from two exposed replicates as well as in groups transferred from sand to mud (shifted) (Table S1). Prior to the weekly water change, the number of TAMs was estimated (MacConnell and Bartholomew 2012). Briefly, water (100 ml) was collected from each container, filtered through a 20-μm mesh filter (Nitex), and the TAMs trapped in the filter were recovered in 10 ml well water. The TAM suspension was mixed gently and aliquoted (100 μl) to a petri dish for total TAM counts using a stereomicroscope (Olympus). This was repeated three times per sample for each 10 ml of suspended filtrate, and a mean TAM value was calculated per 100 ml sampled from each lineage group. The number of TAMs was enumerated and recorded after each sampling up to the end of the study. The parasite amplification ratio is the total number of TAMs produced/total number of myxospores at exposure (Nehring et al. 2014).

Individual TAM release was confirmed from the remaining worms in the non-shifted and shifted groups in sand or mud at the end of the study at 6 months (Table S1, rep1) to validate *M. cerebralis* infection incidence across the lineages particularly for lineage I oligochaetes that harbored the parasite (PCR positive) but negative for parasitic stages (ISH negative) and TAM release. For individual TAM release evaluation, one worm was placed into each 24-well plate (Corning Costar) containing 1 ml well water, incubated for 24–48 h at 12 °C, assessed for TAM presence using a stereoscope (Olympus), and confirmed for genetic lineage.

### Mortality of *Tubifex tubifex* in lineage groups

The number of oligochaetes surviving to the end of the study at 6 months was enumerated from each lineage group (Table S1) that was non-shifted (rep1, rep2) or shifted (rep1) to estimate whether mortalities were more pronounced in susceptible than resistant lineages. The presence of progenies was evaluated across the lineage groups held in sand or mud.

### Analysis of microflora from lineage III *Tubifex tubifex*

Gastrointestinal microflora was assessed in lineage III *Tt* being the most susceptible to *M*. *cerebralis* to determine the potential relationship between the sediment-associated microflora and parasite proliferation. Microflora in oligochaetes was analyzed by Microbial Insights in Rockford, Tennessee, using non-shifted genotype-confirmed lineage III *Tt* (3rd replicate) at 90 days pe to *M. cerebralis* spores in mud (*n* = 3) and sand (*n* = 3) including control (*n* = 3). The *Tt* DNA samples were PCR-amplified to generate different base-pair sequences of mt 16S rDNA fragments and separated by denaturing gradient gel electrophoresis (DGGE) (Muyzer et al. 1993). The DGGE methods and mt 16S rDNA sequencing have been used in gut content analysis of predator species (Suau et al. 1999; Zaidi et al. 1999; Chen et al. 2000). The relative intensity of the bands must constitute at least 1–2% of the total bacterial community to form visible banding patterns for sequencing. The bands were excised, sequenced, and aligned with closely related organisms from GenBank ribosomal database. Phylogenetic affiliations to known organisms in the database were designated by similarity indices: above 0.9 are excellent, 0.7–0.8 are good, and below 0.6 are unique sequences.

### Statistical analysis

The number of TAMs produced in sand or mud in the four *T. tubifex* lineages was analyzed using one-way analysis of variance (ANOVA) followed by Tukey’s honest significant difference (HSD). Differences in TAM production between substrates was assessed separately for each lineage in the non-shifted and shifted groups. These analyses were also used to determine differences in TAM production between lineage I and III regardless of the holding substrate in the non-shifted and shifted groups. Lineages V and VI were not included in the analysis as TAMs were not released in either substrate throughout the study duration. Statistical significance of the tests was set at 5% level, with results considered significantly different when *p* < 0.05.

## Results

### *Tubifex tubifex* lineage typing, *Myxobolus cerebralis* PCR, and ISH

Prior to exposure to *M. cerebralis*, oligochaetes sampled (*N* = 20/lineage group) from the stock cultures to assess for lineage type showed that lineages I and III were homogenous while lineage V contained 80% (16/20) lineage V and 20% (4/20) non-*Tt*; lineage VI contained only 50% (10/20) lineage VI and the rest were lineage V. This lineage profile served as the baseline in the non-shifted (Table 1) and shifted (Table 2) studies. Oligochaetes that lacked any amplified products using the mt 16S rDNA lineage-specific PCR (Beauchamp et al. 2002) were considered non-*Tt*.

**Table 1:** Non-shifted: Lineage composition and detection of *Myxobolus cerebralis* DNA by PCR and in situ hybridization (ISH) in *Tubifex tubifex* lineage groups held continuously in sand or mud until 6 months post exposure (pe) to 1000 myxospores/worm

Lineage—substrate	Lineage^1^ Day 0	Lineage 80 d pe	PCR 80 d pe	ISH 80 d pe	Lineage 6 mo pe	PCR 6 mo pe
I Sand Exposed		I = 9/10^**2**^ **III = 1/10**^**5**^	1/9^**3**^ 1/1	0/9^**3**^ 1/1	I = 20/20^**2**^	0/20^**3**^
I Sand Control	I = 20/20				I = 5/5	0/5
I Mud Exposed		I = 8/10 **III = 2/10**	2/8 2/2	0/8 2/2	I = 14/20 **III = 6/20**	1/14 3/6
I Mud Control					VI = 5/5	0/5
III Sand Exposed		III = 10/10	9/10	9/10	III = 19/20 **VI = 1/20**	10/19 0/1
III Sand Control	III = 20/20				III = 5/5	0/5
III Mud Exposed		III = 10/10	10/10	10/10	III = 10/10	9/10
III Mud Control					III = 5/5	0/5
V Sand Exposed		V = 10/10	0/10	0/10	V = 20/20	0/20
V Sand Control	V = 16/20 Non-Tt = 4/20^4^				V = 5/5	0/5
V Mud Exposed		V = 7/10 Non-Tt = 3/10	0/7 0/3	0/7 0/3	V = 20/20	0/20
V Mud Control					V = 5/5	0/5
VI Sand Exposed		V = 6/10 **III = 2/10**	0/6 0/2	0/6 0/2	VI = 4/20 **V = 16/20**	0/4 0/16
VI Sand Control	VI = 10/20 **V = 10/20**				VI = 5/5	0/5
VI Mud Exposed		VI = 10/10	0/10	0/10	VI = 5/20 **V = 15/20**	0/5 0/15
VI Mud Control					VI = 5/5	0/5

^1^Lineage type at pre-exposure, worms were sampled from stock cultures and examined for lineage type prior to distribution to sediment groups and exposure to *M. cerebralis*

^2^Number of oligochaetes with corresponding lineage/number of oligochaetes examined at 80 days or 6 months

^3^Number of oligochaetes positive for *M. cerebralis* by PCR or ISH/number of worms examined at 80 days or 6 months

^4^Non-*T. tubifex*: absence of amplified products using the 16S *T. tubifex* mitochondrial primers (Beauchamp et al. 2002)

^5^Number of different lineage type(s)/number of oligochaetes examined within the group

**Table 2:** Shifted: Lineage composition and detection of *Myxobolus cerebralis* DNA by PCR in *Tubifex tubifex* lineage groups. Oligochaetes (*N* = 100/lineage) were exposed (1000 myxospores/worm) and held in sand for 60 days; thereafter, 50% of the worms from each lineage were transferred from sand to mud and the 50% retained in sand

Lineage—substrate	Lineage^1^ Day 0	Lineage 6 months pe	PCR 6 months pe
I Sand	I = 20/20	I = 6/6^**2**^	0/6^**3**^
I Mud		I = 19/20 **III = 1/20**^**5**^	3/19 0/1
III Sand	III=20/20	III = 10/10	4/10
III Mud		III = 10/10	10/10
V Sand	V=16/20 Non-Tt=4/20^4^	V = 10/10	0/10
V Mud		V = 20/20	0/20
VI Sand	VI=10/20 **V=10/20**	VI = 4/20 **V = 15/20** **III = 1/20**	0/4 0/15 1/1
VI Mud		VI = 7/20 **V = 13/20**	0/7 0/13

^1^Lineage composition at pre-exposure, worms were sampled from stock culture and examined for lineage prior to distribution to sediment groups and exposure to *M. cerebralis*

^2^Number of oligochaetes with corresponding lineage/number of oligochaetes examined at 6 months

^3^Number of oligochaetes positive for *M. cerebralis* by PCR/number of worms examined at 6 months

^4^Non-*T. tubifex*: absence of amplified products using the 16S *T. tubifex* mitochondrial primers (Beauchamp et al. 2002)

^5^Number of different lineage type(s)/number of oligochaetes examined

At 80 days pe in the non-shifted groups, results for lineage type and *M. cerebralis* screening from the 3rd exposed replicate (*n* = 10) are shown in Table 1. Parasite stages for lineages I and III are shown in Table S2. In the lineage I-sand experimental treatment, 1 of 9 lineage I worms was PCR positive, but all 9 were ISH negative. In contrast, the single lineage III worm was positive by PCR and ISH screening for early parasite stages. Within the lineage I-mud treatment group, 2 of 8 lineage I worms were PCR positive, but all 8 were ISH negative. The two lineage III worms screened in this treatment group were both positive for the PCR and ISH tests. Only lineage III worms were present within the lineage III non-shifted sand and mud experimental treatments. All were PCR positive, and parasite stages were mostly developmental in mud while early to developmental in sand as confirmed by ISH except for one lineage III in sand that was PCR negative and ISH negative for parasite stages. For the resistant lineages, lineage V-sand contained all lineage V worms; lineage V-mud contained all lineage V except for three worms that were non-*Tt*. Lineage VI-sand showed diverse lineage types: V (*n* = 6), VI (*n* = 1), III (*n* = 2), and one non-*Tt*, while lineage VI mud was completely homogenous. Oligochaetes that typed as *Tt* lineage V and VI were all negative by PCR and ISH (Table 1; Table S3).

At 6 months pe in the non-shifted groups, only lineage typing and *M. cerebralis* PCR were conducted (*n* = 20 worms/lineage; Table 1). Lineage I-sand were all lineage I (*n* = 20), and all were *M. cerebralis* negative by PCR. Among the lineage I-mud treatment group, 1 of 14 worms was PCR positive while 3 of 6 worms in that group typed as lineage III were PCR positive for *M. cerebralis* DNA. Among the lineage III-sand treatment group, 10 of 19 were PCR positive while the single lineage VI worm was PCR negative. In the lineage III-mud treatment group, only 10 lineage III worms survived and all, but one was PCR positive. All 20 worms in the lineage V sand and mud treatment groups were PCR negative. For lineage VI-sand treatment, all four lineage VI worms and all 16 lineage V worms were PCR negative. For lineage VI-mud treatment, 5 typed as lineage VI, 15 typed as lineage V, and all were PCR negative. The control unexposed oligochaetes (*n* = 5/lineage group) in sand or mud were all negative for *M. cerebralis* DNA by PCR, and lineage types were homogenous except for oligochaetes in lineage I mud that typed as lineage VI (Table 1).

Lineage typing for *Tt* and *M. cerebralis* PCR was conducted in the shifted study at 6 months (Table 2). In lineage I-sand, the 6 surviving worms typed as lineage I and all were PCR negative. In the lineage I mud treatment, only 3 of 19 lineage I worms were PCR positive, while the single lineage III worm tested PCR negative. Lineage III worms in the sand and mud treatments were homogenous; however, only 4/10 in sand were PCR positive compared to mud where 10 of 10 were PCR positive. For the lineage V treatments in sand (*N* = 10) and mud (*N* = 20), all worms typed as lineage V, and all were negative by PCR. Lineage VI-sand contained mixed lineages: VI (4/20), V (15/20), and III (1/20). All were PCR negative except for the single lineage III worm. Lineage VI-mud contained VI (7/20) mixed with V (13/20), and all were PCR negative.

### TAM production in non-shifted groups

In the non-shifted study, TAM release among the lineage I mud replicates began at 80 days pe and continued until 170 days (Table S4). In lineage I sand replicates, TAM production began at 80 days pe and ceased after 100 days. At the end of the study at 6 months, lineage I produced significantly more TAMs in mud (mean = 2622) compared to sand (mean = 100) (*P* = 0.017; Table 3) with higher parasite amplification ratio in mud (23.7) compared to sand (1.8). Note however that the TAMs released in lineage I groups could be from lineage III oligochaetes present in lineage I group (Table 1).

**Table 3:** Effect of substrate on the production of triactinomyxon spores (TAMs) in *Tubifex tubifex* lineages after exposure to *Myxobolus cerebralis* (1000 spores/worm)

Lineage Non-shifted^1^	Mean number of TAMs		*p*-value^4^	Parasite amplification ratio^5^
I^3^	Sand	100	0.017	Sand = 1.8 Mud = 23.7
	Mud	2622		
III	Sand	2167	0.111	Sand = 19.5 Mud = 49.0
	Mud	5444		
V	Sand	0	**‒**	**‒**
	Mud	0		
VI	Sand	0	**‒**	**‒**
	Mud	0		
Lineage/Shifted^2^				
I^3^	Sand	200	0.036	Sand = 1.8 Mud = 12.0
	Mud	1333		
III	Sand	4556	0.253	Sand = 41.0 Mud = 70.8
	Mud	7867		
V	Sand	0	**‒**	**‒**
	Mud	0		
VI	Sand	0	**‒**	**‒**
	Mud	0		

^1^Non-shifted: Groups (50 worms/lineage) were held continuously in sand or mud for 6 months. TAM numbers represent the mean of two replicates of TAM counts beginning at 80 days post exposure and every 10 days thereafter until 6 months

^2^Shifted: Oligochaetes (*N* = 100/lineage) were exposed to myxospores and held in sand for 60 days; thereafter, 50% of the worms from each lineage were transferred from sand to mud and the rest retained in sand. TAM numbers represent the mean of two replicates of TAM counts beginning at 80 days post exposure and every 10 days thereafter until 6 months

^3^Susceptible lineage III phenotypes were present in lineage I non-shifted (Table 2) and shifted (Table 3) groups that most likely produced the TAMs

^4^Statistical significance is at 5% level; results are significantly different when *p* < 0.05

^5^Parasite amplification ratio = total TAMs produced/total number of myxospores at exposure

Lineage III oligochaetes produced high TAM numbers in sand and mud between 80 and 125 days, which continued to 140 days in mud (Table S4). Although differences in TAM numbers were not significant (*P* = 0.111; Table 3) at the end of the study at 6 months, lineage III in mud produced 2.5-fold more TAMs (mean = 5444) than worms in sand (mean = 2167) with greater parasite amplification in mud (49.0) than sand (19.5).

Lineages V and VI did not release any TAMs in sand or mud (Table 3) at 80 days or at any time up to 6 months pe to *M. cerebralis* (Table S4). The control groups of each lineage (I, III, V, VI) in mud or sand did not produce any TAMs (data not shown).

At the end of the study, TAM release from individual *Tt* (Table 5) was confirmed from non-shifted lineage III: 35.7% (10/28) in sand and 50% (15/30) in mud. Lineage I did not release any TAMs in sand (0/20) and mud (0/33). Lineages V and VI were not confirmed for individual TAM release as the parasite was not detected at 80 days or at any time up to 6 months from all samples examined by PCR and ISH (Tables 1 and 2).

### TAM production in shifted groups

In the shifted study, lineage I retained in sand released TAMs between 80 and 200 days (Table S5). At the end of the study at 6 months, lineage I shifted to mud produced significantly more TAMs (mean = 1333) compared to group retained in sand (mean = 200) (*P* = 0.036) with higher parasite amplification ratio in group transferred to mud (12.0) compared to group retained in sand (1.8) (Table 3). Note however that the TAMs in lineage I could have been released from lineage III oligochaetes present in lineage I (Table 2).

Lineage III worms generally released high TAM numbers whether retained in sand or transferred to mud (Table 3) between 80 and 200 days (Table S5), but differences in TAM numbers were not significant in lineage III (*P* = 0.253) at the end of the study. However, the group shifted to mud produced 1.7-fold more TAMs (mean = 7867) than worms retained in sand (mean = 4556) with higher parasite amplification ratio in mud (70.8) than sand (41.0) (Table 3).

Lineages V and VI did not release any TAMs in groups retained in sand or shifted to mud (Table 3) at 80 days or at any time up until 6 months pe to *M. cerebralis* (Table S5). Shifting the resistant lineages V and VI from sand to mud did not promote the development of any parasite stages (Table 2).

At the end of the study, TAM release from individual *Tt* in the shifted experiment (Table 5) was confirmed from lineage III: 40% (12/30) in sand and 100% (16/16) in mud. Lineage I did not release any TAMs in sand (0/6) and mud (0/48). No TAMs were ever observed in exposed replicates of lineage V and VI oligochaetes (Table 3) in either the non-shifted (Table S4) or shifted experimental groups (Table S5), nor was any parasite DNA detected among the 80 worms (non-shifted) and 69 worms (shifted) screened by PCR at the end of the study (Tables 1 and 2).

### Effect of lineage on TAM production and substrate on *Myxobolus cerebralis* development

Regardless of the holding substrate, lineage type influenced greater TAM production in lineage III than I in the non-shifted (*P* = 0.042) and shifted (*P* = 0.001) groups while lineage type did not impact genotype V and VI on TAM production or lack of TAM release during the study (Table 4).

**Table 4:** Effect of lineage on the production of triactinomyxons (TAMs) in *Tubifex tubifex* at 6 months post exposure to *Myxobolus cerebralis* (1000 spores/worm)

	Mean number of TAMs^1^	*p*-value^3^
Non-shifted—lineage		
I^2^	1361	0.042
III	3806	
V	0	
VI	0	
Shifted—lineage		
I^2^	766	0.001
III	6,212	
V	0	
VI	0	

^1^Average number of TAMs produced from oligochaetes in sand and mud in each lineage group (Table S4; Table S5)

^2^Susceptible lineage III phenotypes were present in lineage I non-shifted (Table 1) and shifted (Table 2) groups that most likely produced the TAMs

^3^Statistical significance is at 5% level, and results are significantly different when *p* < 0.05

**Fig. 1:**
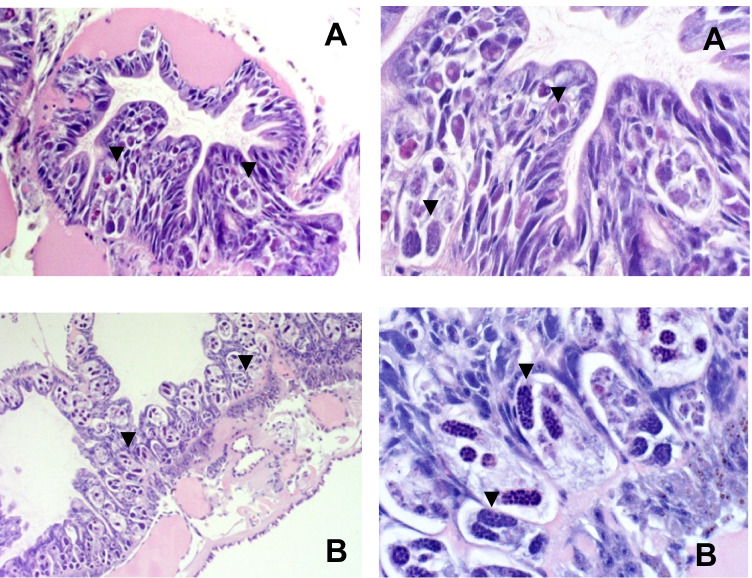
Developmental stages of *Myxobolus cerebralis* in the intestinal mucosa of the oligochaete *Tubifex tubifex* lineage III at 80 days following exposure to 1000 myxospores/worm and holding in sand (**A**) or mud (**B**). Hematoxylin and eosin-stained tissue sections showing early developmental stages (**A**, arrows) in oligochaetes held in sand while oligochaetes kept in mud showed dominant mature stages such as presporogonic to sporogonic forms and nearly mature triactinomyxon spores (**B**, arrows)

Development of *M. cerebralis* in lineage III *Tt* occurred in mud or sand substrate; however, mud better supported sporogenesis compared to oligochaetes in sand that showed early to developmental parasitic forms (Figure 1; Table S2). At 6 months pe, eight of 10 lineage I *Tt* in mud in the non-shifted (Table 1) and 19 of 20 in the shifted (Table 2) studies were PCR positive for parasite DNA. However, at 80 days pe, parasite stages were not detected by ISH in lineage I *Tt* in mud that were positive for *M. cerebralis* by PCR, and parasite DNA was not detected by PCR or ISH in lineage I *Tt* held in sand at 80 days pe (Table S2). Substrate had no impact on lineage V and VI oligochaetes in non-shifted (Table 1) or shifted (Table 2) groups including lack of parasite stages (ISH at 80 days pe; Table S3). All lineage V and VI *Tt* remained entirely negative for *M. cerebralis* DNA (Tables 1 and 2; Table S3) hence the lack of TAM release (Tables 3 and 4; Table S4; Table S5).

### Mortality of *Tubifex tubifex* across lineages in sand and mud

In the non-shifted study, the most susceptible lineage III oligochaetes showed generally high mortalities in both substrate: 51% in sand and 54% in mud based on the mean of the two exposed replicates. Lineage I showed greater mortalities in sand (60%) than mud (17%). The resistant lineage V had minimal deaths in sand (7%) compared to mud (41%). Lineage VI worms in sand experienced 77% mortality compared to 26% in mud (Table 5). In the shifted groups, high mortalities were also observed in lineage III in sand (40%) and mud (68%). Lineage I oligochaetes had 88% mortality in sand compared to 4% in mud. Refractory lineage V worms retained in sand experienced 28% mortality but no mortalities in mud. Among the lineage VI worms, mortality retained in sand was 20% compared to 56% for those retained in mud. Progeny was produced by all lineage groups shifted to mud compared to lack of progeny in groups retained in sand except for the lineage VI group. In contrast, progeny production was highly variable among groups held continuously in the same substrate (Table 5).

**Table 5:** Individual release of triactinomyxon spores (TAMs) from and mortality profile of *Tubifex tubifex* in sand or mud at 6 months post exposure to *Myxobolus cerebralis* (1000 spores/worm). The number of adult (a) and progeny (p) was enumerated at the end of the study at 6 months

Lineage—substrate	Non-shifted^1^				Control	Mortality
	Exposed 1	Mortality	Exposed 2	Mortality		
I Sand	20a (0/20)^**3**^; 4p	30 (60%)	20a; 12p	30 (60%)	20a; 1p	30 (60%)
I Mud	33 (0/33)	17 (34%)	106 (+56 p)	0	150 (+100 p)	0
III Sand	28a (10^+^/28); 16p	22 (44%)	21a; 28p	29 (58%)	21a; 15p	29 (58%)
III Mud	30a (15^+^/30); 55p	20 (40%)	16a	34 (68%)	60p (+10)	0
V Sand	46a; 7p^**4**^	4 (8%)	47a; 3p	3 (6%)	34a; 28p	16 (32%)
V Mud	28a	22 (44%)	31a	19 (38%)	35	15 (30%)
VI Sand	12a; 36p^**4**^	38 (76%)	11a;10p	39 (78%)	30a; 62p	20 (40%)
VI Mud	24	26 (52%)	50	0	60 (+10)	0
	Shifted^**2**^	Mortality				
I Sand	6 (0/6)^**3**^	44 (88%)	na^**5**^		na	
I Mud	48a (0/48); 60p	2 (4%)	na		na	
III Sand	30 (12^+^/30)	20 (40%)	na		na	
III Mud	16a (16^+^/16); 50p	34 (68%)	na		na	
V Sand	36a^**4**^	14 (28%)	na		na	
V Mud	100a; 50p	0	na		na	
VI Sand	40a; 80p^**4**^	10 (20%)	na		na	
VI Mud	22a; 180p	28 (56%)	na		na	

^1^Non-shifted: Groups (50 worms/lineage) were continuously held in sand or mud substrates until the end of the study at 6 months. Individual TAM release was assessed only from the first replicate group of exposed oligochaetes in sand or mud

^2^Shifted: Groups (*N* = 100 worms/lineage) were held in sand for 60 days after which 50% of the worms were shifted to mud and the rest retained in sand. Individual TAM release was assessed from one exposed group of oligochaetes in sand or mud as replicates were not established in the shifted experiment

^3^Number of worms positive for TAM release over the number of worms individually screened in 24-well culture plates to assess for TAM production. Individual worms were plated in 24-well plates with 1 ml well water, incubated for 24–48 h at 12 °C, and assessed for TAM release using a stereoscope

^4^Lineages V and VI oligochaetes were not assessed for individual TAM release as parasite stages were not detected in all samples examined in the non-shifted and shifted groups in sand or mud at 80 days and 6 months pe (Tables 1 and 2), and TAMs were not released in exposed lineage V and VI groups of non-shifted and shifted groups in either substrate (Table 3)

### Analysis of microflora from lineage III *Tubifex tubifex*

The DGGE profiles of lineage III *Tt* held in sand or mud showed different intensities of banding patterns (Figure S1). The recovered bands allowed the comparison of microflora sequences from lineage III *Tt* to sequence data of known organisms in GenBank (Table S6). Several genera were commonly found in lineage III *Tt* in either substrate including *Flavobacterium, Leptotrichia*, *Bacteriovax*, and *Zoogloea*. Some species were unique in lineage III *Tt* sand such as *Blastochloris*, *Pseudomonas*, and *Rhizobium* including species belonging to Rhodocyclaceae and Rhodobacteraceae while *Helicobacter* and *Treponema* were found only in lineage III *Tt* in mud (Table S6).

## Discussion

Rearing the *Tt* lineage groups in sand or mud following laboratory exposure to *M. cerebralis* allowed the assessment of substrate effects to *Tt* genotype on parasite proliferation and release of the infectious waterborne TAMs. Lineage III *Tt* held in mud better supported parasite development to sporogenesis. Triactinomyxon (TAM) release was 2.5-fold greater in mud than sand in both studies held in the substrate continuously (non-shifted) or with substrate changes from sand to mud (shifted). Individual TAM release at the end of the study showed that all lineage III *Tt* (16/16) transferred from sand to mud released TAMs compared to only 40% (12/30) in lineage III *Tt* retained in sand. Holding the lineage III *Tt* in the same substrate showed relatively decreased TAMs although still higher in mud (50% = 15/30) than sand (35% = 10/28) (Table 5). These results corroborate our initial findings in pure clonal populations of lineage III that were altered from non-TAM producing (resistant) to TAM-producing (susceptible) phenotypes following transfer from sand to mud (Baxa et al. 2006, 2008). Our previous and current results indicate that susceptible *Tt* are more likely to amplify TAM production in mud compared to coarse substrates and are consistent with the findings of others (Arndt et al. 2002; Blazer et al. 2003). At point sources or “hot spots” where high densities of susceptible *Tt* hosts and salmonid fish are sympatric in aquatic ecosystems, the risk of *M. cerebralis* proliferation can intensify in fine organic sediments and exacerbate the extent and severity of WD infections among susceptible salmonid species.

Substrate did not influence *M. cerebralis* development in lineage I *Tt*. Although ca.15% of lineage I in mud were *M. cerebralis* PCR positive, parasitic stages were not detected by ISH (Table S2). Furthermore, individual TAM release was never detected among genotype-confirmed lineage I *Tt* in the non-shifted and shifted studies (Table 5) in contrast to lineage III *Tt* that consistently released TAMs until the end of the study (Table 5; Table S4; Table S5). Additionally, mature sporogonic and TAM stages commonly released into the gut lumen and then expelled from the worm (El-Matbouli and Hoffmann 1998; Hedrick et al. 1998; Hedrick and El-Matbouli 2002) were not observed in lineage I *Tt* in our study. These results indicate that the TAMs produced in lineage I in sand or mud (Table 3) were most likely released from the lineage III worms present in the group (Tables 1 and 2). The few lineage III worms that produced high numbers of TAMs in the lineage I replicates could be debatable. However, previous laboratory studies have documented that TAM production by a single infected *Tt* oligochaete can range as high as 46,000 in 23 days (Gilbert and Granath 2001). In another laboratory exposure, 250 lineage III *Tt* exposed to only 50 *M. cerebralis* myxospores/worm produced a total of 22.49 million TAMs at 180 days pe (Nehring et al. 2014). In mixed lineages (I, III, IV, and VI) of *Tt* exposed to 500 myxospores/worm, only lineage III *Tt* were infected with *M. cerebralis* (qPCR) where only 1 of 158 surviving worms (0.6%) was infected, and it was 1 of 7 surviving lineage III *Tt* at the end of the study (Arsan et al. 2007a).

Resistance to *M. cerebralis* or disparity in TAM production has been well documented in lineage I *Tt* across geographic regions. Exposure to various doses of myxospores did not result in any TAM release in lineage I oligochaetes from Alaska (Arsan et al. 2007a), the Willamette River, Oregon (Hallett et al. 2009), the San Juan River, New Mexico (DuBey and Caldwell 2004), the Gallatin River drainage in Montana (Kerans et al. 2004, 2005) or the Eagle and Gunnison rivers in Colorado (Nehring et al. 2014). In the current study, exposure to 1000 myxospores/worm for the lineage I strain from Colorado was adequate for assessing substrate effects across the lineage groups as shown in the reference lineage III phenotypes that produced an average (per worm) of 533 TAMs in sand and 1125 TAMs in mud. In contrast, although the lineage I oligochaetes showed a low prevalence of infection by *M. cerebralis* PCR in either substrate, the non-shifted and shifted groups failed to release any TAMs; hence, lineage I *Tt* are considered resistant in this study.

Substrate did not influence genotypes V and VI that remained refractory to *M. cerebralis* regardless of the rearing substrate or changes in substrate type. Our results corroborate a body of evidence that lineages V and VI are not susceptible to infection with *M. cerebralis* as TAM release has never been observed following exposure to the parasite (Beauchamp et al. 2005; DuBey et al. 2005; Steinbach-Elwell et al. 2006; Hallett et al. 2009; Nehring et al. 2014). As biological filter, *Tt* lineage V and VI can ingest, remove, and deactivate myxospores preventing their development to the mature actinosporean TAM stage infectious to susceptible young salmonids thus decreasing overall infectivity (El-Matbouli et al 1999a, b; Beauchamp et al. 2005, 2006; Nehring et al. 2016, 2018). It is unknown whether lineage I–resistant strains could act as biological filter by ingesting and inactivating myxospores as shown in lineage V and VI *Tt* (El-Matbouli et al. 1999a; Nehring et al. 2016). It is possible that sporoplasms from *M. cerebralis* myxospores ingested by lineage I *Tt* invaded the intestinal mucosa but failed to develop and vanished (El-Matbouli et al. 1999a) or the parasite developed but arrested as shown in lineage III–resistant strain (Baxa et al. 2008). Both theories may, in part, explain the presence of the parasite DNA (PCR positive) but absence of parasite stages (ISH negative) in lineage I strains following exposure to *M. cerebralis* (Table 1; Table S2) despite the type of the holding substrate.

Mortality was most pronounced in susceptible lineage III oligochaetes that showed the most severe infections with *M. cerebralis* in sand or mud. In contrast, the non-susceptible lineages V and VI and resistant lineage I oligochaetes showed disparate survival in the presence or absence of exposure to the parasite in either substrate. While the experimental conditions may have affected the overall fitness of the oligochaetes, environmental effects on *Tt* lineages are unknown (Dubey et al. 2005). High mortalities in lineage III oligochaetes could be directly caused by severe infections with *M. cerebralis* that can negatively impact the growth, reproduction, and survival of susceptible *Tt* hosts (El-Matbouli and Hoffmann 1998; Stevens et al. 2001; Hedrick and El-Matbouli 2002; Gilbert and Granath 2003). It is interesting to note that juvenile oligochaetes were present in all lineage groups shifted to mud in contrast to the lack of progeny in groups held continuously in the same substrate. Interpretation of this finding is ambiguous; however, the substrate-associated microbial community in the mud may have provided adequate food sources or that the absence of the parasite in the new (mud) substrate enhanced nutritional efficiency and fecundity of the *Tt* host (Shirakashi and El-Matbouli 2009).

The substrate-associated microbial community in lineage III phenotypes contained diverse bacterial species associated with predation by *Tt* (de Valk et al. 2019). However, we were unable to determine the potential relationship between the bacterial community in the substrate and *M. cerebralis* development in the *Tt* host due to the small sample size of oligochaetes used for microbial analysis. We hypothesize that the resident bacteria in the mud provided a vital dietary source to the worm host as shown in benthic sediment predators (Hargrave 1970). Although *Tt* feed selectively on silt-clay and associated organic materials (Rodriguez et al. 2001), substrate selection is not determined solely by particle size but mainly on the microflora associated with the substrata (Wavre and Brinkhurst 1971; McMurtry et al. 1983). The *Tt* host continuously ingests not only bacteria and decayed organic materials found in refined sediments but also *M. cerebralis* spores (Hedrick et al. 1998; Granath and Gilbert 2002) that adhere well in fine sediments compared to coarse particles (Lemmon and Kerans 2001). Ingestion of myxospores (Hedrick et al. 1998; Lemmon and Kerans 2001; Granath and Gilbert 2002) and other microflora (Wavre and Brinkhurst 1971; McMurtry et al. 1983) by *Tt* is more apt to occur in mud due to their preference for small particles (Rodriguez et al. 2001). In free-flowing streams, the small size of *M. cerebralis* myxospores (≤ 10 µm diameter) allows them to be mobilized in the water column and carried by the current to settle in back-water eddies where the water velocity is low or zero. These areas of the stream are also areas where fine organic material settle, providing an ideal microhabitat and substrate for decomposers of organic material such as bacteria and aquatic oligochaetes. Together, these factors may, in part, explain the proliferation of *M. cerebralis* in the oligochaete host in mud and less so in sand, facilitating parasite development and TAM production in the mud substrate in our study. Previous studies (Lemmon and Kerans 2001; Arndt et al. 2002, Blazer et al. 2003; Krueger et al. 2006) lend support to our findings that mud, compared to sand, enhances parasite development in susceptible *Tt*. Nevertheless, further investigations using large sample size of *Tt* genotypes in natural habitats may provide insights on the relationship between the invertebrate host, their associated microbiome, and the role that the sediment-associated microflora may play in promoting *M. cerebralis* propagation and WD severity.

Our study underscores the role of substrate as a factor contributing to *M. cerebralis* development and proliferation in susceptible *Tt* genotypes. The co-occurrence of resistant lineage I and susceptible lineage III oligochaetes (Tables 1 and 2) did not inhibit parasite development (Table S2) and TAM release in the susceptible lineage III (Table 3). Steinbach-Elwell et al. (2006) and Hallett et al. (2009) also showed that resistant strains did not reduce *M. cerebralis* proliferation and infection prevalence in susceptible strains. Our results support their findings. However, our study demonstrates that organically rich fine sediments can alter parasite infectivity in susceptible *Tt* strain by enhancing sporogenesis and TAM release. In Colorado where certain sites are the epicenter of WD, Beauchamp et al. (2005) and subsequently Nehring et al. (2016) demonstrated that the severity of WD hinges on the genetic composition of *Tt* populations that are sympatric with wild trout populations susceptible to *M. cerebralis* infection. Although their studies did not emphasize substrate type, their findings showed that WD is more severe when susceptible lineages are more abundant while the predominance of resistant lineage V or VI, including more resistant lineage I, may reduce WD severity or disrupt the life cycle of *M. cerebralis* (Nehring et al. 2016, 2018).

Understanding the extent of substrate effect in altering *M. cerebralis* infectivity in susceptible *Tt* hosts may help to manage WD risk when the pathogen and the invertebrate host overlap in environments that can be mitigated to reduce the influence of substrate on the spread and impact of WD. Coarse sediments that are less favorable for development of robust populations of *Tt* hosts can reduce the proliferation of *M. cerebralis* or even prevent the establishment of the parasite. Invertebrate hosts of myxozoans have been targeted for potential management of diseases that they cause; however, many invertebrates have yet to be discovered on their capacity to host or sustain parasite life cycles (Fontes et al. 2015). Our work augments habitat data that may aid in determining the role of substrate on diminishing the effects of WD on wild trout populations through mitigation of environments to combat the development of *M. cerebralis* in susceptible *Tt* hosts.

## Supplementary Information

Below is the link to the electronic supplementary material.

Supplementary file1 (DOCX 185 KB)

## Data Availability

The data that were generated or analyzed during this study are included in this article and its supplementary information files. If further clarification is needed, requests may be directed to the corresponding author.
