# Supplementary material for: Effect of substrate on the proliferation of Myxobolus cerebralis in the mitochondrial lineages of the Tubifex tubifex host
Source: Parasitol Res. 2022 Jul 27;121(9):2503–16. doi: 10.1007/s00436-022-07587-4 (PMC9378325; doi:10.1007/s00436-022-07587-4)
Supplement: Supplementary file 1 — Supplementary file1 (DOCX 185 KB) [file 436_2022_7587_MOESM1_ESM.docx]

**Supplementary Table S1** Schematic design of non-shifted and shifted studies in *Tubifex tubifex* lineages after exposure to *Myxobolus cerebralis* (1,000 myxospores/worm) and maintained in mud or sand


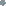


| **Non-shifted^1^** | Sand for 6 months | | | | Mud for 6 months | | | |
| --- | --- | --- | --- | --- | --- | --- | --- | --- |
| Lineage | Control | Replicate-1 | Replicate-2 | Replicate-3 | Control | Replicate-1 | Replicate-2 | Replicate-3 |
| I | 50 | 50 | 50 | 50 | 50 | 50 | 50 | 50 |
| III | 50 | 50 | 50 | 50 | 50 | 50 | 50 | 50 |
| V | 50 | 50 | 50 | 50 | 50 | 50 | 50 | 50 |
| VI | 50 | 50 | 50 | 50 | 50 | 50 | 50 | 50 |

Control

• Lineage typing prior to

myxospore exposure, N=20

• PCR *M. cerebralis*, 6 months,

N=10

• Mortality, 6 months

Replicate-3

• Lineage typing, 80 days N=10,

6 months N=20

• PCR *M. cerebralis*, 80 days N=10,

6 months N=20

• ISH *M. cerebralis*, 80 days N=10

Replicate-1/Replicate-2

• No sampling

• TAM counts, weekly up to 6 months

• TAM release/worm (Rep-1), 6 months,

N=all survivors

• Mortality, 6 months

| **Shifted^2^**  Lineage | Held in sand  for 60 days | Retained in sand  at 60 days | Shifted from sand to mud  at 60 days |
| --- | --- | --- | --- |
| I | 100 | 50 | 50 |
| III | 100 | 50 | 50 |
| V | 100 | 50 | 50 |
| VI | 100 | 50 | 50 |

Groups retained in sand/shifted to mud

• Lineage typing, 6 months, N=20

• *M. cerebralis* PCR, 6 months, N=20

• TAM release/worm, 6 months, N=all survivors

• Mortality, 6 months

Exposed groups/lineage in sand or mud

• No sampling for 60 days

**^1^**Non-shifted: Groups of 50 worms/lineage were held continuously in sand or mud for 6 months. Water samples were collected from replicates 1 and 2 for assessing the number of triactinomyxon spores (TAMs) released into the water; replicate 1 survivors at 6 months were used for individual TAM release. Oligochaetes (N=10) were sampled from replicate-3 at 80 days post exposure (pe) for examination of *T. tubifex* lineage type and presence of *M. cerebralis* DNA by PCR and in situ hybridization (ISH). At termination of the study at 6 months pe, remaining worms (N=up to 20 or all survivors) from replicate-3 were used for lineage typing and *M. cerebralis* PCR.

**^2^**Shifted: Exposed groups of 100 worms/lineage were held in sand for 60 days thereafter 50% of the worms were shifted from sand to mud and the remaining 50% were retained in sand. Lineage typing and *M. cerebralis* PCR were conducted at termination of the study at 6 months in groups retained in sand and in groups shifted to mud (N=up to 20 or all survivors).

**Supplementary Table S2** Non-shifted: Lineage composition and detection of *Myxobolus cerebralis* DNA in *Tubifex tubifex* lineage I and III as determined by PCR and in situ hybridization (ISH) at 80 days post exposure to 1,000 myxospores/worm

| Lineage  Substrate | Lineage type | *M. cerebralis*  PCR | *M. cerebralis*  ISH/stages | Lineage  Substrate | Lineage  type | *M. cerebralis*  PCR | *M. cerebralis*  ISH/stages |
| --- | --- | --- | --- | --- | --- | --- | --- |
| **I Sand** |  |  |  | **I Mud** |  |  |  |
| 1 | I | ‒ | ‒ | 1 | III | + | + developmental**^2^** |
| 2 | III | + | + early**^1^** | 2 | III | + | + developmental |
| 3 | I | + | ‒ | 3 | I | ‒ | ‒ |
| 4 | I | ‒ | ‒ | 4 | I | ‒ | ‒ |
| 5 | I | ‒ | ‒ | 5 | I | ‒ | ‒ |
| 6 | I | ‒ | ‒ | 6 | I | + | ‒ |
| 7 | I | ‒ | ‒ | 7 | I | + | ‒ |
| 8 | I | ‒ | ‒ | 8 | I | ‒ | ‒ |
| 9 | I | ‒ | ‒ | 9 | I | ‒ | ‒ |
| 10 | I | ‒ | ‒ | 10 | I | ‒ | ‒ |
| **III Sand** |  |  |  | **III Mud** |  |  |  |
| 1 | III | ‒ | ‒ | 1 | III | + | + developmental |
| 2 | III | + | + developmental | 2 | III | + | + early |
| 3 | III | + | + developmental | 3 | III | + | + early |
| 4 | III | + | + developmental | 4 | III | + | + developmental |
| 5 | III | + | + developmental | 5 | III | + | + developmental |
| 6 | III | + | + early | 6 | III | + | + developmental |
| 7 | III | + | + developmental | 7 | III | + | + developmental |
| 8 | III | + | + early | 8 | III | + | + developmental |
| 9 | III | + | + developmental | 9 | III | + | + developmental |
| 10 | III | + | + early | 10 | III | + | + developmental |

Indicates the presence (+) or absence (‒) of *M. cerebralis* DNA by PCR or parasite stages by ISH; H&E-stained sections were paired with ISH sections for screening of *M. cerebralis* stages.

**^1^**Early merogonic stages of *M. cerebralis* present in *T. tubifex* section as determined by ISH.

**^2^**Developmental includes mixed parasite stages: late merogonic, presporogonic, and sporogonic forms in *T. tubifex* section as determined by ISH.

**Supplementary Table S3** Non-shifted: Lineage composition and detection of *Myxobolus cerebralis* DNA in *Tubifex tubifex* lineage V and VI as determined by PCR and in situ hybridization (ISH) at 80 days post exposure to 1,000 myxospores/worm

| Lineage  Substrate | Lineage type | *M. cerebralis*  PCR | *M. cerebralis*  ISH/stages | Lineage  Substrate | Lineage  type | *M. cerebralis*  PCR | *M. cerebralis*  ISH/stages |
| --- | --- | --- | --- | --- | --- | --- | --- |
| **V Sand** |  |  |  | **V Mud** |  |  |  |
| 1 | V | ‒**^1^** | ‒ | 1 | Non-Tt**^2^** | ‒ | ‒**^1^** |
| 2 | V | ‒ | ‒ | 2 | Non-Tt | ‒ | ‒ |
| 3 | V | ‒ | ‒ | 3 | V | ‒ | ‒ |
| 4 | V | ‒ | ‒ | 4 | Non-Tt | ‒ | ‒ |
| 5 | V | ‒ | ‒ | 5 | V | ‒ | ‒ |
| 6 | V | ‒ | ‒ | 6 | V | ‒ | ‒ |
| 7 | V | ‒ | ‒ | 7 | V | ‒ | ‒ |
| 8 | V | ‒ | ‒ | 8 | V | ‒ | ‒ |
| 9 | V | ‒ | ‒ | 9 | V | ‒ | ‒ |
| 10 | V | ‒ | ‒ | 10 | V | ‒ | ‒ |
| **VI Sand** |  |  |  | **VI Mud** |  |  |  |
| 1 | V | ‒ | ‒ | 1 | VI | ‒ | ‒ |
| 2 | V | ‒ | ‒ | 2 | VI | ‒ | ‒ |
| 3 | V | ‒ | ‒ | 3 | VI | ‒ | ‒ |
| 4 | VI | ‒ | ‒ | 4 | VI | ‒ | ‒ |
| 5 | III | +**^1^** | +early**^3^** | 5 | VI | ‒ | ‒ |
| 6 | V | ‒ | ‒ | 6 | VI | ‒ | ‒ |
| 7 | V | ‒ | ‒ | 7 | VI | ‒ | ‒ |
| 8 | III | ‒ | ‒ | 8 | VI | ‒ | ‒ |
| 9 | Non-Tt | ‒ | ‒ | 9 | VI | ‒ | ‒ |
| 10 | V | ‒ | ‒ | 10 | VI | ‒ | ‒ |

**^1^**Indicates the presence (+) or absence (‒) of *M. cerebralis* DNA by PCR or parasitic stages by ISH; H&E-stained sections were paired with ISH sections for screening of *M. cerebralis* stages.

**^2^**Non-*Tubifex tubifex*, no amplification using the *T. tubifex* specific primers (Beauchamp et al. 2001) and verified with the mt 16S rDNA lineage-specific PCR (Beauchamp et al. 2002).

**^3^**Early merogonic stages of *M. cerebralis* present in *T. tubifex* section as determined by ISH.

**Supplementary Table S4** Non-shifted: Number of triactinomyxon spores (TAMs) produced from *Tubifex tubifex* lineages (N=50 worms/lineage) after exposure to *Myxobolus cerebralis* (1,000 myxospores/worm) and held continuously in sand or mud substrate for six months

| **Days post exposure** | **Lineage I**^1^ | | | | **Lineage III** | | | | **Lineage V^2^** | | **Lineage VI^2^** | |
| --- | --- | --- | --- | --- | --- | --- | --- | --- | --- | --- | --- | --- |
|  | Sand1 | Sand2 | Mud1 | Mud2 | Sand1 | Sand2 | Mud1 | Mud2 | Sand | Mud | Sand | Mud |
| 80 | 0 | 1,200 | 200 | 0 | 6,000 | 3,000 | 10,000 | 6,000 | 0 | 0 | 0 | 0 |
| 90 | 0 | 200 | 2,400 | 1,400 | 11,000 | 8,000 | 14,000 | 10,000 | 0 | 0 | 0 | 0 |
| 100 | 0 | 400 | 2,600 | 1600 | 2,000 | 4,000 | 4,000 | 10,000 | 0 | 0 | 0 | 0 |
| 115 | 0 | 0 | 6,000 | 3,000 | 1,000 | 2,000 | 3,000 | 14,000 | 0 | 0 | 0 | 0 |
| 125 | 0 | 0 | 2,400 | 12,000 | 600 | 600 | 10,000 | 12,000 | 0 | 0 | 0 | 0 |
| 140 | 0 | 0 | 1,200 | 10,000 | 600 | 0 | 600 | 2,000 | 0 | 0 | 0 | 0 |
| 160 | 0 | 0 | 2,000 | 0 | 200 | 0 | 0 | 600 | 0 | 0 | 0 | 0 |
| 170 | 0 | 0 | 2,400 | 0 | 0 | 0 | 1,200 | 600 | 0 | 0 | 0 | 0 |
| 200 | 0 | 0 | 0 | 0 | 0 | 0 | 0 | 0 | 0 | 0 | 0 | 0 |
| Total number of TAMs | 0 | 1800 | 19,200 | 28,000 | 21,400 | 17,600 | 42,800 | 55,200 | 0 | 0 | 0 | 0 |
| Mean number of TAMs | 0 | 200 | 2,133 | 3,111 | 2,378 | 1,956 | 4,756 | 6,133 | 0 | 0 | 0 | 0 |

**^1^**Susceptible lineage III phenotypes were present in lineage I non-shifted groups (Table 1, Table S2) that most likely produced the TAMs.

**^2^**Lineages V and VI had two replicate groups of exposed oligochaetes that were held in sand and mud that did not release any TAMs.

Note: Control groups for each lineage that were not exposed to myxospores did not develop any parasitic stages and did not release any TAMs in sand or mud (data not shown).

**Supplementary Table S5** Shifted: Number of triactinomyxon spores (TAMs) produced from *Tubifex tubifex* lineages after exposure to *Myxobolus cerebralis* (1,000 myxospores/worm). Groups (N=100/lineage) were held in sand for 60 days thereafter 50% of the worms were shifted from sand to mud and the 50% retained in sand

| **Days post exposure to sand** | **Days post transfer to mud** | **Lineage I^1^** | | **Lineage III** | | **Lineage V** | | **Lineage VI** | |
| --- | --- | --- | --- | --- | --- | --- | --- | --- | --- |
|  |  | **Sand** | **Mud** | **Sand** | **Mud** | **Sand** | **Mud** | **Sand** | **Mud** |
| 80 | 20 | 200 | 800 | 10,000 | 20,000 | 0 | 0 | 0 | 0 |
| 90 | 30 | 0 | 1600 | 12,000 | 17,000 | 0 | 0 | 0 | 0 |
| 100 | 40 | 400 | 4,000 | 5,000 | 6,000 | 0 | 0 | 0 | 0 |
| 115 | 55 | 0 | 2,000 | 3,000 | 10,000 | 0 | 0 | 0 | 0 |
| 125 | 65 | 0 | 1,200 | 4,000 | 12,000 | 0 | 0 | 0 | 0 |
| 140 | 80 | 0 | 0 | 2,000 | 2,000 | 0 | 0 | 0 | 0 |
| 160 | 100 | 600 | 0 | 3,000 | 3,000 | 0 | 0 | 0 | 0 |
| 170 | 110 | 0 | 2,400 | 1,200 | 800 | 0 | 0 | 0 | 0 |
| 200 | 140 | 600 | 0 | 800 | 0 | 0 | 0 | 0 | 0 |
| Total number of TAMs |  | 1,800 | 12,000 | 41,000 | 70,800 | 0 | 0 | 0 | 0 |
| Mean number of TAMs |  | 200 | 1,333 | 4,556 | 7,867 | 0 | 0 | 0 | 0 |

**^1^**Susceptible lineage III phenotypes were present in lineage I shifted groups (Table 2) that most likely produced the TAMs.

**Supplementary Table S6** Microbial composition in *Tubifex tubifex* lineage III in sand and mud as determined by denaturing gradient gel electrophoresis and mt 16S rDNA sequencing. Species identification is based on DNA sequences in GenBank. Similarity indices above 0.9 are excellent, 0.7-0.8 are good, and below 0.6 are considered unique sequences

| Band/  Sediment | Genus | Similarity Index | GenBank Accession Number |
| --- | --- | --- | --- |
| 1.1 Control Mud | *Leptotrichia* | 0.74 | AY029802; AF287816 |
| 1.2 Control Mud | *Bacteriovorax* | 0.65 | AY947967 |
| 2.1 Control Mud | *Flavobacterium* | 0.78 | AF502206 |
| 2.2 Control Mud | *Leptotrichia* | 0.86 | AF287816; AY548984 |
| 3.2 Control Mud | *Bacteriovorax* | 0.65 | AY947967 |
| 4.1 Exposed Mud | ***Helicobacter*^1^** | 0.97 | AY631954; M35048; M88156 |
| 4.2 Exposed Mud | *Flavobacterium* | 0.73 | DQ298761, DQ298763 |
| 4.4 Exposed Mud | ***Treponema*^1^** | 0.81 | AY340818 |
| 5.1 Exposed Mud | *Treponema* | 0.82 | AY340818 |
| 5.2 Exposed Mud | *Zoogloea* | 0.93 | AY212743 |
| 6.1 Exposed Mud | *Helicobacter* | 0.93 | AY034820 |
| 6.2 Exposed Mud | *Flavobacterium* | 0.72 | DQ298761 |
| 6.3 Exposed Mud | *Leptotrichia* | 0.86 | AY548984 |
| 7.1 Control Sand | *Zoogloea* | 0.59 | AY676493; AY928246; AF011344 |
| 7.2 Control Sand | *Zoogloea* | 0.58 | AY676493; AY928246; AF011344 |
| 8.1 Control Sand | *Flavobacterium* | 0.73 | AM114447; AM114448; AM403312 |
| 8.3 Control Sand | *Leptotrichia* | 0.86 | AY548984 |
| 8.4 Control Sand | ***Blastochloris*^2^** | 0.90 | AJ012089; AY117150; D25314 |
| 9.1 Control Sand | *Zoogloea* | 0.59 | AY676493; AY928246; AF011344 |
| 9.2 Control Sand | *Zoogloea* | 0.59 | AY676493; AY928246; AF011344 |
| 10.2 Exposed Sand | *Bacteriovorax* | 0.63 | AY947967 |
| 10.4 Exposed Sand | **Rhodocyclaceae^2^** | 0.82 | AY212743 |
| 11.1 Exposed Sand | ***Pseudomonas*^2^** | 1.00 | AY689078; AY663434 |
| 11.2 Exposed Sand | *Pseudomonas* | 0.78 | AY382613 |
| 11.3 Exposed Sand | *Bacteriovorax* | 0.65 | AY947967 |
| 11.4 Exposed Sand | **Rhodobacteraceae^2^** | 0.86 | AY913410 |
| 11.5 Exposed Sand | ***Rhizobium*^2^** | 1.00 | X74915 |
| 12.1 Exposed Sand | *Zoogloea* | 0.59 | AY676493; AY928246; AF011344 |
| 12.2 Exposed Sand | *Bacteriovorax* | 0.65 | AY947967 |
| 12.4 Exposed Sand | *Zoogloea* | 0.92 | AY212743 |

**^1^***Helicobacter* and *Treponema* are unique in lineage III-mud.

**^2^***Blastochloris*, Rhodocyclaceae, *Pseudomonas*, Rhodobacteraceae, and *Rhizobium* are unique in lineage III-sand. All other genera are common to oligochaetes in mud and sand.


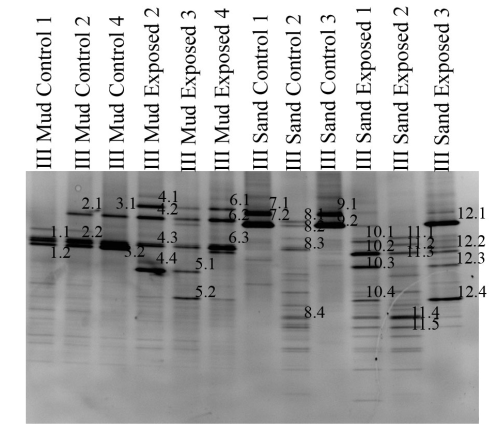


**Supplementary Figure S1** Denaturing gradient gel electrophoresis (DGGE) profile of amplified DNA from a portion of the mt 16S rRNA gene of lineage III *Tubifex tubifex* in sand and mud substrate at 90 days post exposure to *Myxobolus cerebralis*. Relative intensities of the bands must constitute at least 1-2% of the total bacterial community to form visible banding patterns for sequencing.
